# Obesity-Associated Gestational Diabetes Promotes Cellular Heterogeneity and Dysfunction in Neonatal Offspring-Islets

**DOI:** 10.3390/nu18030464

**Published:** 2026-01-30

**Authors:** Xiangju Cao, Jian Wang, Xinyu Jia, Shuai Yang, Yuan Wang, Lixia Ji

**Affiliations:** 1 Department of Pharmacology, School of Pharmacy, Qingdao University, Qingdao 266071, China; cxj19863348170@163.com (X.C.); jiaxinyuzzz@163.com (X.J.); yangbb09252024@163.com (S.Y.); wangyuanqdu@163.com (Y.W.); 2 Department of Laboratory Animal Science, School of Medicine, Shanghai Jiao Tong University, Shanghai 200025, China; 184305@shsmu.edu.cn

**Keywords:** GDM, offspring, islet development, single-cell RNA sequencing, β-cell dysfunction

## Abstract

**Background/Objectives**: Given the lack of clarity regarding how maternal overnutrition during pregnancy regulates offspring metabolic health, our study intends to explore the specific influences of maternal Western diet (WD) exposure on neonatal islet cell development and heterogeneity. **Methods**: Using a WD-induced gestational diabetes mellitus (GDM) rat model, we assessed glucose homeostasis via blood glucose and serum insulin levels. Target protein expression and islet function were evaluated using immunofluorescence and insulin secretion assays, respectively. To delineate alterations in cellular heterogeneity, we subsequently performed single-cell RNA sequencing (scRNA-seq) on isolated islet cells. **Results**: Maternal WD exposure induced significant glucose intolerance and insulin resistance, confirming GDM establishment. Their neonatal offspring consequently displayed disrupted glucose homeostasis, characterized by concurrent hypoglycemia, hyperinsulinemia, and enhanced insulin secretion. ScRNA-seq analysis further identified the enhanced endocrine cells in GDM-offspring islets, with imbalanced α/β-cell subsets—specifically, reduced immature α1/β1 subsets and expanded mature α2/β2/β3/β4 subsets, alongside upregulated expression of insulin- and glucagon-related genes (Ins1, Ins2, Gcg). Notably, β cells in GDM offspring displayed metabolic hyperactivity (enriched ribosomal and glycolytic pathways) with multiple organelle dysfunction, including mitochondrial swelling, cristae reduction, decreased membrane potential, and severe endoplasmic reticulum stress. **Conclusions**: The metabolic dysregulation of WD-induced GDM in maternal rats is transmitted to offspring, leading to disrupted neonatal α/β-cell subset balance and accelerated islet maturation. However, such excessive development comes at the cost of organelle damage in β cells. Our findings provide a molecular basis for mitigating the intergenerational transmission of diabetes through early nutritional interventions.

## 1. Introduction

Metabolic disorders, with diabetes at the forefront, are gradually evolving into a global epidemic. The westernization of dietary patterns and sedentary lifestyles, which are risk factors in this epidemic, have led to excessive energy accumulation in the body [1,2,3]. However, in addition to these acquired factors, growing evidence suggests that the root causes of metabolic diseases may be traced back to early life, with maternal nutritional status during pregnancy emerging as a critical determinant [4]. The “Developmental Origins of Health and Disease (DOHaD)” theory points out that an adverse intrauterine microenvironment can trigger “developmental reprogramming” of fetal organs, and this impact has a memory effect, which is one of the fundamental causes for the high incidence of metabolic diseases in offspring during adulthood [5].

The relationship between diabetes and pregnancy is mainly classified into two categories. The first is pregestational diabetes mellitus (PGDM), which refers to diabetes diagnosed before conception and mainly includes type 1 and type 2 diabetes. The second is gestational diabetes mellitus (GDM), defined as glucose metabolism disorders that are first onset or identified during pregnancy. These two conditions present distinct pathological profiles. In PGDM, significant hyperglycemia and associated metabolic derangements are typically preexisting, often leading to a decompensated regulatory state in key metabolic organs. Conversely, GDM usually develops in the second or third trimester. Most individuals with GDM are in a prediabetic or early diabetic stage, where the body remains in an active compensatory state yet is frequently burdened by prominent glucolipid toxicity and low-grade metabolic inflammation. According to the 11th edition of the International Diabetes Federation Diabetes Atlas, the global incidence of hyperglycemia in pregnant women is approximately 19.7%, affecting about 23 million newborns. Among these, 79.2% are attributable to GDM, indicating that GDM has a broader impact on offspring [6].

Excessive weight gain during early pregnancy has been identified as a significant risk factor for GDM. The westernization of dietary structure (characterized by excessive energy density, insufficient dietary fiber, and imbalanced macronutrient ratios) is one of the primary inducing factors for GDM. It not only impairs maternal health but also exerts long-term impacts on offspring metabolic function via the aforementioned metabolic memory effect [7,8,9]. Both epidemiological and clinical studies have confirmed that GDM offspring face a significantly higher risk of developing obesity, insulin resistance, and type 2 diabetes (T2D) in adulthood [10,11,12]. While GDM offspring share comparable postnatal living and nutritional conditions with healthy counterparts, their embryonic development occurs in a markedly altered intrauterine nutritional and metabolic microenvironment. Women with GDM progressively develop a microenvironment characterized by hyperglycemia, hyperlipidemia, and low-grade inflammation from the second trimester onward, and these abnormalities worsen as gestation advances [13]. It is under this hypermetabolic stress that GDM offspring complete their tissue and organ development, as well as partial functional maturation.

As the core endocrine organ regulating glucose homeostasis, islets are highly sensitive to the intrauterine energetic metabolic microenvironment. The period from late embryogenesis to the neonatal stage represents a critical window for the development, differentiation, and functional maturation of islet cells [14]. Disruption of intrauterine nutrient balance during this window can induce islet developmental reprogramming, leading to ultrastructural and functional abnormalities of islet cells, which constitute a key cause of the increased incidence of glucose metabolic disorders in adult offspring. Our previous work has revealed that that in a microenvironment of maternal hyperglycemia or overnutrition, the islet development of their offspring mice is overactivated in the late embryonic stage [15,16,17]. Additionally, studies in high-fat diet (HFD)-induced PGDM models showed marked cellular heterogeneity in fetal acinar and β cells. Reduced insulin secretion coincides with compensatory overexpression of key transcription factors like Pdx1, indicating adaptive cellular remodeling under nutritional and metabolic imbalance stress [18]. However, the changes in cellular heterogeneity and the molecular mechanisms underlying the abnormal development of islet cells in offspring induced by maternal overnutrition during pregnancy remain incompletely defined.

Using a Western diet (WD)-induced GDM rat model, this study systematically characterized neonatal islet abnormalities in offspring through molecular biology, single-cell RNA sequencing (ScRNA-seq), and morphological approaches. By clarifying the mechanism through which maternal nutritional perturbation-driven GDM microenvironment affects the development and function of offspring islet cells, these findings lay a theoretical foundation for early nutritional interventions to mitigate the intergenerational transmission of diabetes. 

## 2. Materials and Methods

### 2.1. GDM Rat Model

Sprague–Dawley rats were purchased from Beijing Vital River Lab Animal Technology Co., Ltd. (SCXK-2021-0011, Beijing, China). All rats were maintained under laboratory conditions (with the temperature kept at 22–25 °C and a 12 h light/dark cycle). All animal experimental procedures were approved by the Animal Ethics Committee of Qingdao University (Approval No.: 20230401SD15020241130196). A GDM model was established as previously described [19]. Briefly, 24 ten-week-old female rats were used in the experiment and divided into two groups. The GDM group received a WD (41% kcal from fat, 42.5% kcal from carbohydrate, 16.5% kcal from protein; HFK Ltd., Beijing, China) starting one week prior to mating and continuing until parturition, while the normal control (NC) group and male rats were fed a standard diet (10% kcal from fat, 70% kcal from carbohydrate, 20% kcal from protein; HFK Ltd.).

To control for potential confounding effects of litter size on neonatal metabolic outcomes, litters with fewer than 8 or more than 15 pups were excluded from this study. Only litters containing 10–13 pups were included for subsequent analyses.

### 2.2. Oral Glucose Tolerance Test (OGTT)

Pregnancy was confirmed via vaginal smears following overnight mating of female and male rats at a ratio of 2 females to 1 male. The concurrent presence of cornified epithelial cells and sperm was defined as embryonic day 0.5 (E0.5). At E14.5, an OGTT was performed on pregnant rats to identify the GDM model. In this process, after a 4 h fast, fasting blood glucose levels in each group were measured (recorded as 0 min values). A 50% (*w*/*v*) glucose solution was then administered via gavage at a dose of 2 g/kg body weight, with blood glucose levels recorded at 30, 60, and 120 min after glucose loading.

### 2.3. Serum Insulin Detection

Blood samples were collected from maternal rats and their offspring at postnatal day 0 (P0) for insulin quantification using the Ultrasensitive Rat Insulin ELISA kit (10–1251-01, Mercodia, Uppsala, Sweden) according to the manufacturer’s instructions.

### 2.4. Immunofluorescence (IF)

Fetal pancreas samples were promptly dissected and fixed in 4% paraformaldehyde for 24–48 h, after which they were subjected to paraffin embedding using standard procedures. In addition, 4 μm paraffin sections were prepared within one week. Then, these sections were blocked with 10% goat serum and incubated overnight at 4 °C with primary antibodies, including anti-insulin (Cat# ab282459, 1:1000, Abcam, Cambridge, UK), and anti-glucagon (Cat# NBP2-21803F, 1:4000, Novus Biologicals, Centennial, CO, USA). The next day, sections were incubated with the corresponding secondary antibodies. Finally, immunofluorescence images of the target protein were captured using a laser confocal microscope (Nikon, Tokyo, Japan).

### 2.5. Transmission Electron Microscope (TEM)

The pancreata of P0 offspring rats were promptly fixed in 2.5% glutaraldehyde, followed by ethanol dehydration, resin embedding, sectioning, and counterstaining. Finally, the ultrastructure of islet cells was examined using a transmission electron microscope. Two blinded observers independently scored mitochondrial morphology (0–3) based on matrix expansion and cristae integrity: 0 (normal), 1 (mild swelling, slight expansion), 2 (moderate swelling, partial cristae disruption), 3 (severe swelling, complete cristae loss/vacuolation). Interobserver agreement was confirmed (weighted Kappa = 0.761). The mean score per animal served as the individual datapoint for intergroup statistical analysis.

### 2.6. Primary Islet Isolation

After anesthetization, the abdomen was opened to dissect the pancreas, which was rinsed with PBS buffer containing 1% penicillin-streptomycin and subsequently cut into small pieces. 1 mL of pre-cooled collagenase P solution (Cat# 11213865001, 0.5 mg/mL, Roche, Basel, Switzerland) was then added, followed by incubation at 38 °C for 5–10 min with gentle pipetting until a fine slurry formed. Digestion was stopped by adding Hanks solution with 10% fetal bovine serum (FBS). The mixture was centrifuged at 4 °C, 1000 rpm for 3 min, and the cell pellet was resuspended in culture medium supplemented with 10 μL of DTZ solution. After allowing the suspension to stand for 2 min, the islets, which had stained scarlet, were carefully hand-picked under a microscope. These isolated islets were then inoculated into RPMI1640 medium containing 10% FBS in preparation for subsequent experiments.

### 2.7. ScRNA-Seq

The islets isolated as described above were incubated with Dispase II (GC26118, 6 U/mL; Glpbio, Montclair, CA, USA) at 37 °C for 3–5 min to digest them into single cells. Subsequently, the primary islet cells were transferred to CapitalBio Technology Co., Ltd. (Beijing, China). for the following steps, including cell lysis, nucleic acid capture, mRNA reverse transcription and amplification, library construction, and sequencing.

### 2.8. Insulin Secretion In Vitro

Islets of similar size and morphology were selected and seeded into 12-well plates at 50 islets per well. The glucose- and amino acid-stimulated insulin secretion assay was performed as previously described [17]. Briefly, after starvation in Krebs Ringer Bicarbonate (KRB) buffer containing 2.8 mM glucose, the cells were sequentially incubated for 60 min in fresh KRB buffers with 2.8 mM glucose, 2.8 mM glucose + 1 μmol/mL total amino acid mixture (AA), or 16.7 mM glucose. The supernatants were collected separately, and insulin secretion levels were measured using the Ultrasensitive Rat Insulin ELISA kit (10–1251-01, Mercodia, Sweden). Subsequently, total protein content of the islets was quantified using a BCA protein assay kit, and the measured insulin levels were normalized to the total protein content.

### 2.9. Determination of Mitochondrial DNA (mtDNA) Copy Number

DNA from primary islet cells of each group was extracted using the StarSpin Universal DNA Kit (D133-01, GenStar, Beijing, China). Subsequently, based on the principle of quantitative real-time PCR (qPCR) amplification, the relative mitochondrial-to-reference gene copy number ratio was calculated by comparing their Ct values to determine relative mtDNA levels. qPCR was performed as described above [19], and the relative copy number of the mitochondrial gene (CYTB) was normalized to the Rn18s rRNA gene using the 2^−ΔΔCt^ method. Only Ct values between 15 and 30 without non-specific amplification were included in the analysis. The primer sequences of CYTB and Rn18s rRNA are as follows:
m-mt-CYTB-F ACCTCCTATCAGCCATCCCATA;m-mt-CYTB-RGAAGAGGAGGTGAACGATTGCT;m-Rn18s-F CGCCGCTAGAGGTGAAATTC;m-Rn18s-R CCAGTCGGCATCGTTTATGG. 

### 2.10. Mitochondrial Membrane Potential (ΔΨm) Detection

Consistent with manufacturer specifications, the JC-1 probe (C2006, Beyotime Biotechnology, Shanghai, China) was utilized to assess ΔΨm dynamics. High ΔΨm triggered JC-1 aggregation into J-aggregates within the mitochondrial matrix, emitting red fluorescence. Conversely, ΔΨm dissipation caused probe dissociation into cytoplasmic monomers, generating green fluorescence. Confocal imaging captured these electrochemical transitions spatially (Leica, Wetzlar, Germany). Additionally, ΔΨm was quantified by flow cytometry. Briefly, cells were stained with JC-1, incubated at 37 °C, washed, and then subjected to flow cytometric analysis (Beckman Coulter, Brea, CA, USA). The fluorescence intensity of JC-1 aggregates and monomers was measured, and their ratio was calculated.

### 2.11. Statistical Analysis

Data are presented as the mean ± SEM. For the animal experimental data, all statistical analyses were conducted using GraphPad Prism 10.0 software (Boston, MA, USA), with the unpaired two-tailed Student’s *t*-test applied for statistical analysis. *p*-value < 0.05 was considered statistically significant.

## 3. Results

### 3.1. WD-Induced Maternal Rats Exhibit a GDM Phenotype Characterized by Disturbances in Glucose Metabolism and Insulin Resistance

**Figure 1:**
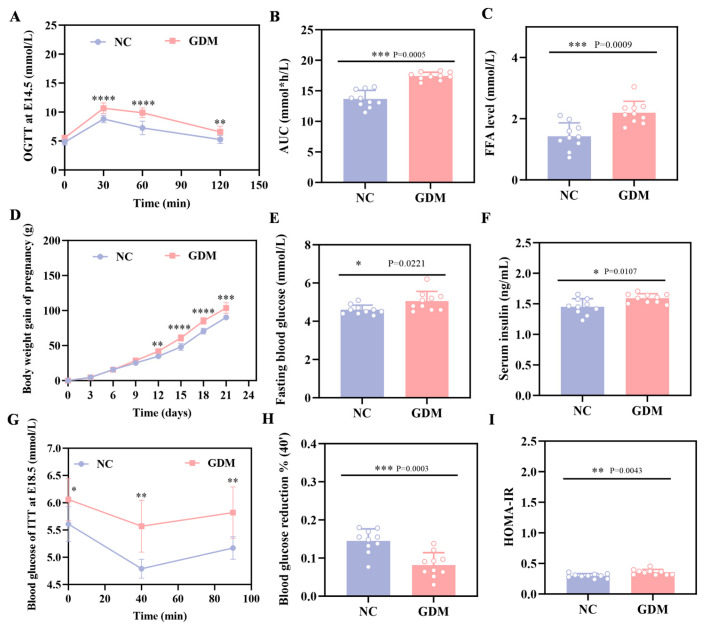
WD induces glucose metabolic dysfunction in pregnant rats. (**A**) Blood glucose levels and (**B**) AUC during OGTT at E14.5. (**C**) FFA level in pregnant at P0. (**D**) Body weight gain during gestation in pregnant rats. (**E**) Fasting blood glucose levels at P0 in pregnant rats. (**F**) Fasting serum insulin levels at P0 in pregnant rats. (**G**) ITT blood glucose and (**H**) percentage of 40 min blood glucose drop at E18.5 in pregnant rats. (**I**) HOMA-IR in pregnant rats at P0 (n = 10). Data were analyzed using unpaired two-tailed Student’s *t* tests. Results are expressed as mean ± SEM, with statistical significance indicated as follows: * *p* < 0.05, ** *p* < 0.01, *** *p* < 0.001, and **** *p* < 0.0001.

The OGTT results in this study showed that WD feeding induced significant hyperglycemia in pregnant rats at E14.5, with blood glucose levels markedly elevated at all time points after glucose load compared to the NC group (*p* < 0.05, Figure 1A). Concomitantly, the area under the blood glucose curve (AUC) was 28% greater than in the NC group (Figure 1B). These rats, meeting GDM diagnostic criteria as a direct consequence of the dietary intervention, established a validated model for subsequent experiments.

As depicted in Figure 1D, WD feeding also affected maternal body weight, with a clear divergence between the GDM and NC groups emerging from E12, and the GDM group exhibiting accelerated weight gain as gestation progressed. Additionally, maternal assessments on P0 showed that the dietary challenge resulted in approximately 1.5-fold higher FFA levels in the GDM group relative to the NC group (Figure 1C). Consistently, WD-induced GDM maternal rats displayed elevated fasting blood glucose levels, accompanied by a compensatory increase in serum insulin (Figure 1E,F). Further calculation of the Homeostatic Model Assessment of Insulin Resistance (HOMA-IR) demonstrated significantly higher values in the GDM group (Figure 1I), underscoring the diet-induced metabolic dysfunction. We also performed an ITT on E18.5 pregnant rats. Data from Figure 1G,H demonstrate that GDM dams had a reduced blood glucose decline at 40 min post-insulin injection and a quicker rebound compared to the NC group. Taken together, these findings indicated that WD feeding successfully generated a maternal GDM phenotype characterized by glucose metabolism disturbances and enhanced insulin resistance.

### 3.2. Maternal WD Exposure Leads to Islet Hyperfunction and Disrupted Glucose Homeostasis in Offspring Rats

**Figure 2:**
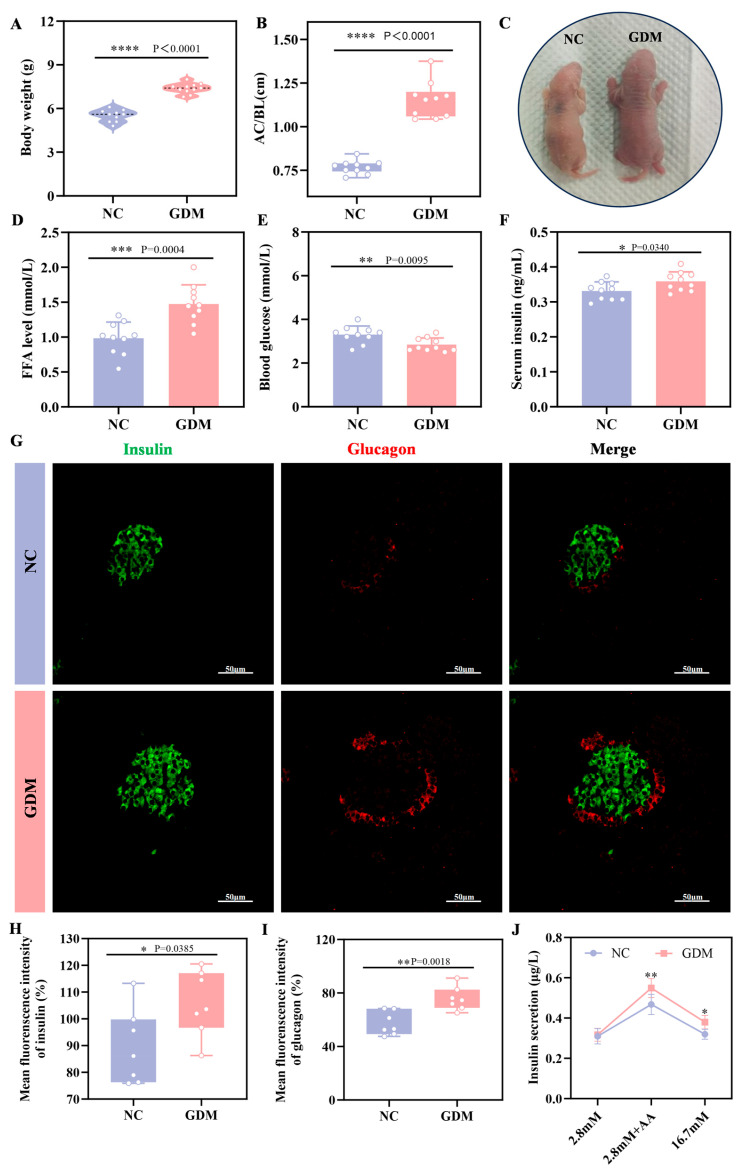
WD-induced GDM triggers islet overdevelopment in offspring rats at P0. (**A**) Body weight of offspring rats (n = 10). (**B**) Abdominal circumference/body length (AC/BL) of offspring rats (n = 10). (**C**) Representative images of offspring rats. (**D**) FFA level of offspring rats. (**E**) Fasting blood glucose levels of offspring rats (n = 10). (**F**) Fasting serum insulin levels of offspring rats (n = 10). (**G**–**I**) Confocal image and quantification of insulin+ cells (green) and Glucagon+ cells (red) in pancreatic sections of the offspring rats (n = 7). Scale bar, 50 μm. (**J**) Insulin secretion levels respond to glucose or amino acids in the primary islets of offspring (n = 3 L). Data were analyzed using unpaired two-tailed Student’s *t* tests. Results are expressed as mean ± SEM, with statistical significance indicated as follows: * *p* < 0.05, ** *p* < 0.01, *** *p* < 0.001, and **** *p* < 0.0001.

To explore the impact of the maternal WD-induced GDM microenvironment on offspring, we examined growth and metabolic indices in P0 offspring rats. The results showed that offspring from WD-fed dams developed significant alterations, including increased body weight and a markedly elevated abdominal circumference to body length ratio (AC/BL), which was approximately 52% higher than that of NC offspring (*p* = 0.0340, Figure 2A–C). Metabolically, these diet-exposed offspring exhibited significant hypoglycemia at birth (*p* = 0.0095, Figure 2E), whereas serum insulin levels were markedly elevated (*p* < 0.0001, Figure 2F). Further analysis of the islet tissue revealed that the level of FFA was also significantly increased in the islets of GDM offspring (Figure 2D). Immunofluorescence analysis (Figure 2G–I) showed that at P0, islets of WD-induced GDM offspring showed significantly increased volume compared to NC group, accompanied by enhanced immunofluorescence intensity of both insulin and glucagon. Sensing glucose and secreting insulin is an essential function of mature islets. As a classic method for evaluating islet function in vitro, insulin secretion assays (Figure 2J) showed no statistical difference in insulin secretion between the two groups under stimulation with 2.8 mM glucose. However, under stimulation with amino acid, insulin secretion in the GDM group was significantly higher than that in the NC group (*p* = 0.00385), and this difference was more pronounced under stimulation with 16.7 mM glucose (*p* = 0.018). Notably, in physiological state, normal neonatal islets are only sensitive to amino acid and not to high-concentration glucose [20]. These results collectively indicated that maternal WD exposure led to the coexistence of significant islet hyperfunction and glucose metabolism disorders in offspring rats at birth, reflecting the abnormal developmental status of their islet function.

### 3.3. Maternal WD Exposure Disrupts Functional Gene Expression in β and α Cells of Offspring Rats

**Figure 3:**
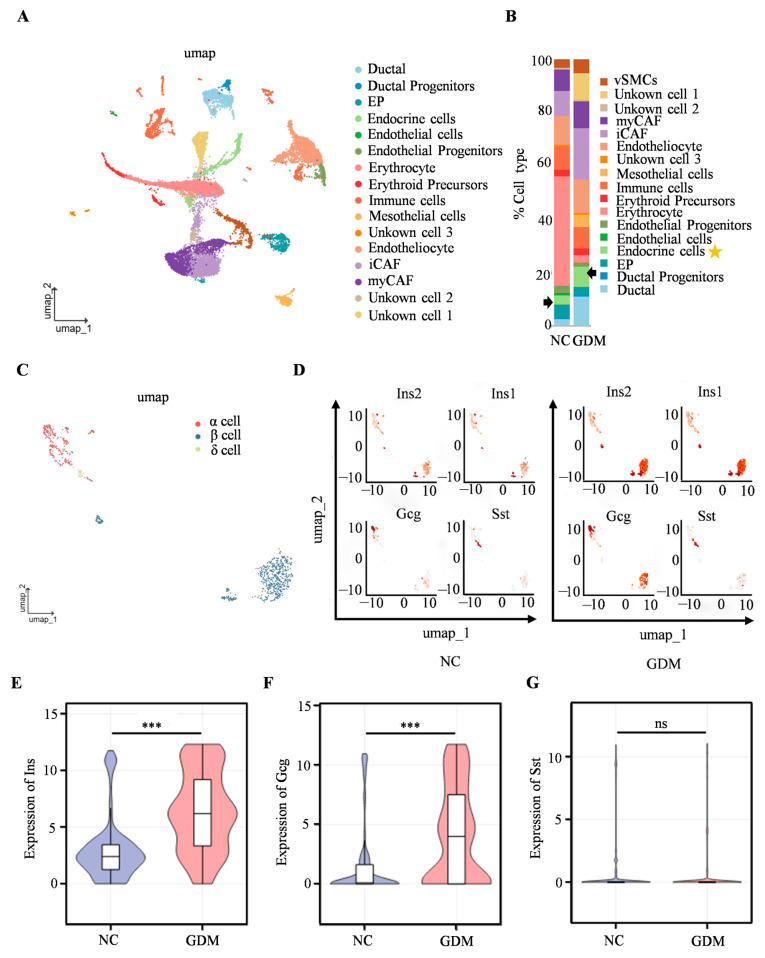
Single-cell transcriptomics reveals islet cellular remodeling in WD-induced GDM-offspring rats at P0. (**A**) UMAP projection of all cells within isolated primary islets. (**B**) Percentage of all cell types. The arrows and asterisks in Figure B indicate endocrine cells. (**C**) UMAP projection of endocrine cells. (**D**) UMAP visualization of Ins, Gcg, and Sst expression in endocrine cells. The axes of the scatter plot correspond to the two-dimensional spatial coordinates generated by the t-SNE/UMAP algorithm, with the color gradient from gray to red indicating an incremental increase in expression level. (**E**–**G**) Expression levels of INS, GCG, and SST in endocrine cells. (n = 3 L). Data were analyzed using unpaired two-tailed Student’s *t* tests. Results are expressed as mean ± SEM, with statistical significance indicated as follows: *** *p* < 0.001. ns: not significant.

To further investigate the mechanism underlying islet dysfunction in WD-induced GDM offspring, scRNA-seq was performed on isolated islets from P0 offspring rats. We identified cell types such as erythrocytes, ductal cells, and endocrine cells (Figure 3A), with the proportion of endocrine cells of WD-induced GDM offspring being significantly higher than that in the NC group (Figure 3B). Subsequent analysis focusing on endocrine cells revealed a predominance of α, β, and δ cells (Figure 3C). The UMAP dimensionality reduction clustering results (Figure 3D) showed that, compared with the NC group, the cell clusters corresponding to the insulin-related genes (Ins2 and Ins1), as well as the glucagon gene (Gcg), in the islet cells of WD-induced GDM offspring exhibited more concentrated distribution and increased cell numbers. In contrast, there was no significant change in the distribution of the cell cluster corresponding to the somatostatin gene (Sst). Furthermore, Ins1, Ins2, and Gcg expression were significantly upregulated in WD-induced GDM-offspring islet cells, while the Sst level remained comparable between groups (Figure 3E–G). These findings suggested that the WD-induced GDM microenvironment may promote the expression of genes related to insulin and glucagon secretion in offspring islets, altering the distribution and functional state of islet cells.

### 3.4. Imbalance of α and β Cell Subsets in Islets of WD-Induced GDM-Offspring Rats

**Figure 4:**
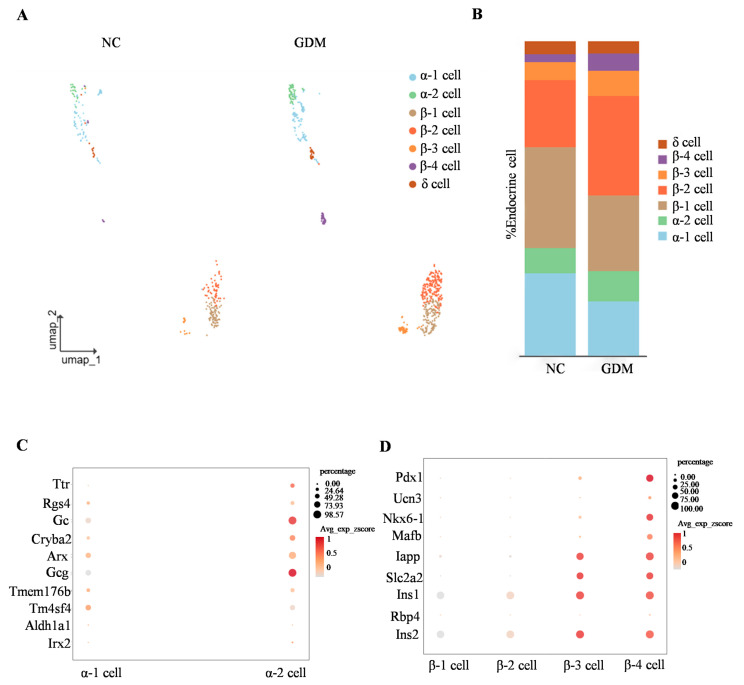
WD-induced GDM causes subset remodeling of α and β cells in offspring rats at P0. (**A**) UMAP projection of all endocrine cells with respective subclusters in the NC- and WD-induced GDM-offspring rat islets. (**B**) Percentage of α, β, and δ cells. (**C**,**D**) Dot plots illustrating key identity gene expression levels in α cell (**C**) and β cell (**D**) subclusters.

By subclassifying islet α, β, and δ cell subsets, we identified that α cells are primarily composed of α1 and α2 subsets, while β cells are mainly divided into four subsets (β1, β2, β3, and β4) (Figure 4A). Analysis of cell proportion differences showed that, compared with the NC group, the proportions of α1 and β1 subsets in the islets of WD-induced GDM-offspring rats were significantly decreased (Figure 4B). In contrast, the proportions of α2, β2, β3, and β4 subsets were increased (Figure 4B).

To clarify the molecular characteristics of α cell subsets, we detected genes related to α cell function (such as Ttr, Rgs4, Gc, and Arx). The results showed that these genes were expressed in both α1 and α2 subsets (Figure 4C). Notably, expression of the glucagon-encoding gene Gcg was significantly higher in the α2 subset, suggesting that the α2 subset may represent a more mature functional phenotype (Figure 4C).

Similarly, gene expression analysis of β-cell subsets showed differential expression patterns in key regulators of functional maturation, including Pdx1, Ucn3, and Nkx6-1 (Figure 4D). Consistent with this, color gradient visualization of average expression z-scores (Avg_exp_zscore) demonstrated that genes governing insulin secretion and glucose sensing (Iapp, Slc2a2, Ins1, Ins2) exhibited progressively elevated expression levels in β3 and β4 subsets (Figure 4D). These findings collectively indicated a spatiotemporal developmental trajectory across β cell subsets, reflecting gradual functional maturation from β1 to β4.

### 3.5. The WD-Induced GDM Microenvironment Upregulates GLP-1, PI3K, and AMPK Signaling in Offspring Islets

Wang et al. have demonstrated that FAM3A deficiency in α cells upregulates islet-derived glucagon-like peptide-1 (GLP-1). The underlying mechanism involves upregulation of NR4A2 expression triggered by FAM3A deficiency. Subsequently, NR4A2 interacts with FOXA2 to form a transcriptional complex that binds to cis-regulatory elements within the PCSK1 promoter, repressing its transcriptional activity and ultimately enhancing GLP-1 biosynthesis in α cells [21].

**Figure 5:**
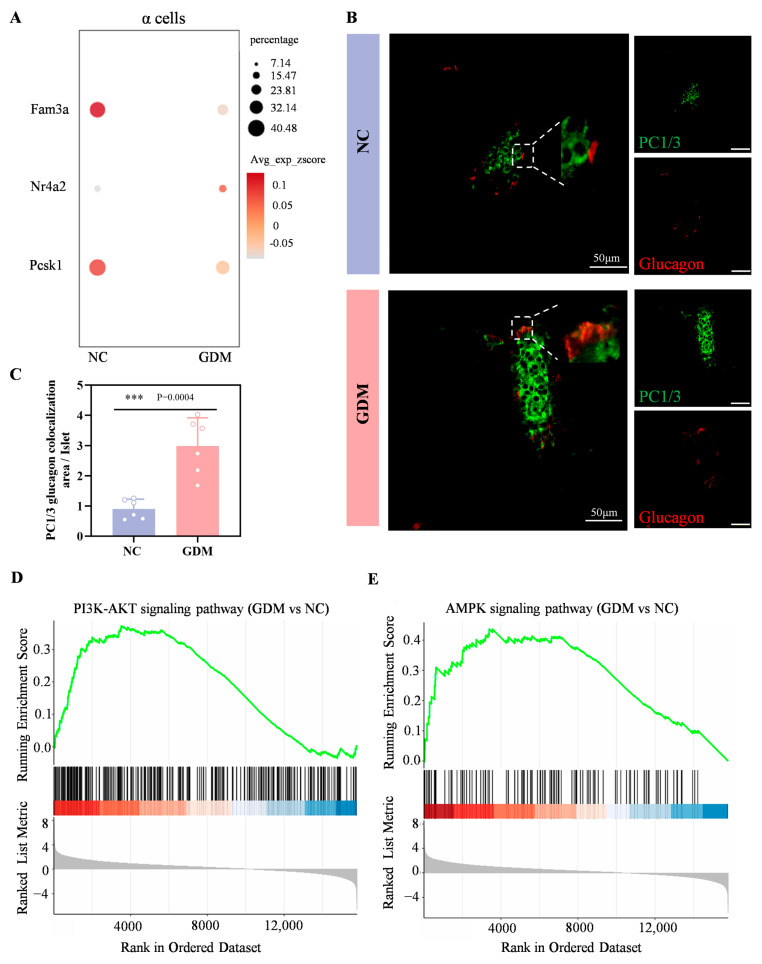
Maternal WD exposure upregulates the GLP-1, PI3K-AKT, and AMPK signaling pathways in offspring islets. (**A**) Dot plots showing the expression levels of Fam3a, Nr4a2, and Pcsk1genes within α cells of islets in the offspring rats at P0. (**B**,**C**) Confocal images and quantification of PC1/3 (green) and glucagon (red) colocalization in pancreatic sections from P0 offspring rats (n = 6). Scale bar, 50 μm. (**D**,**E**) Enrichment plot of the PI3K-AKT (**D**) and AMPK (**E**) signaling pathway in WD-induced GDM versus NC groups. Data were analyzed using unpaired two-tailed Student’s *t* tests. Results are expressed as mean ± SEM, with statistical significance indicated as follows: *** *p* < 0.001.

In the sequencing results of this study, compared with the NC group, the expression levels of FAM3A and PCSK1 genes in α cells of WD-induced GDM-offspring rats were significantly downregulated, while the expression of NR4A2 gene was significantly upregulated (Figure 5A). Immunofluorescence analysis further showed a markedly larger area proportion of PC1/3^+^Glucagon^+^ cells (indicating GLP-1^+^) in the WD-induced GDM group compared with the NC group at P0 (Figure 5B,C). These results suggested that α cells in the islets of WD-induced GDM-offspring rats produce significantly more GLP-1.

As shown in Figure 5D,E, both the PI3K-AKT signaling pathway and the AMPK signaling pathway were significantly upregulated in P0 islets of WD-induced GDM offspring. Numerous studies have shown that early activation of the PI3K–Akt–FoxO1 signaling can promote the development and differentiation of α and β cells [22,23]. Combined with our previous findings that the GLP-1/GLP-1R axis can activate the PI3K–Akt pathway, leading to the activation of FoxO1 and mTORC1, which in turn promote the premature initiation of functional maturation programs in β cells [17,24]. Meanwhile, as a core hub of cellular energy metabolism [25,26,27], activation of the AMPK signaling pathway in islet cells may further synergistically promote islet cell development by regulating energy-sensing status, enhancing prohormone processing efficiency, or affecting transcription factor activity.

### 3.6. WD-Induced GDM Causes Metabolic Hyperactivity and Organelle Dysfunction in β Cells of Offspring Rats

**Figure 6:**
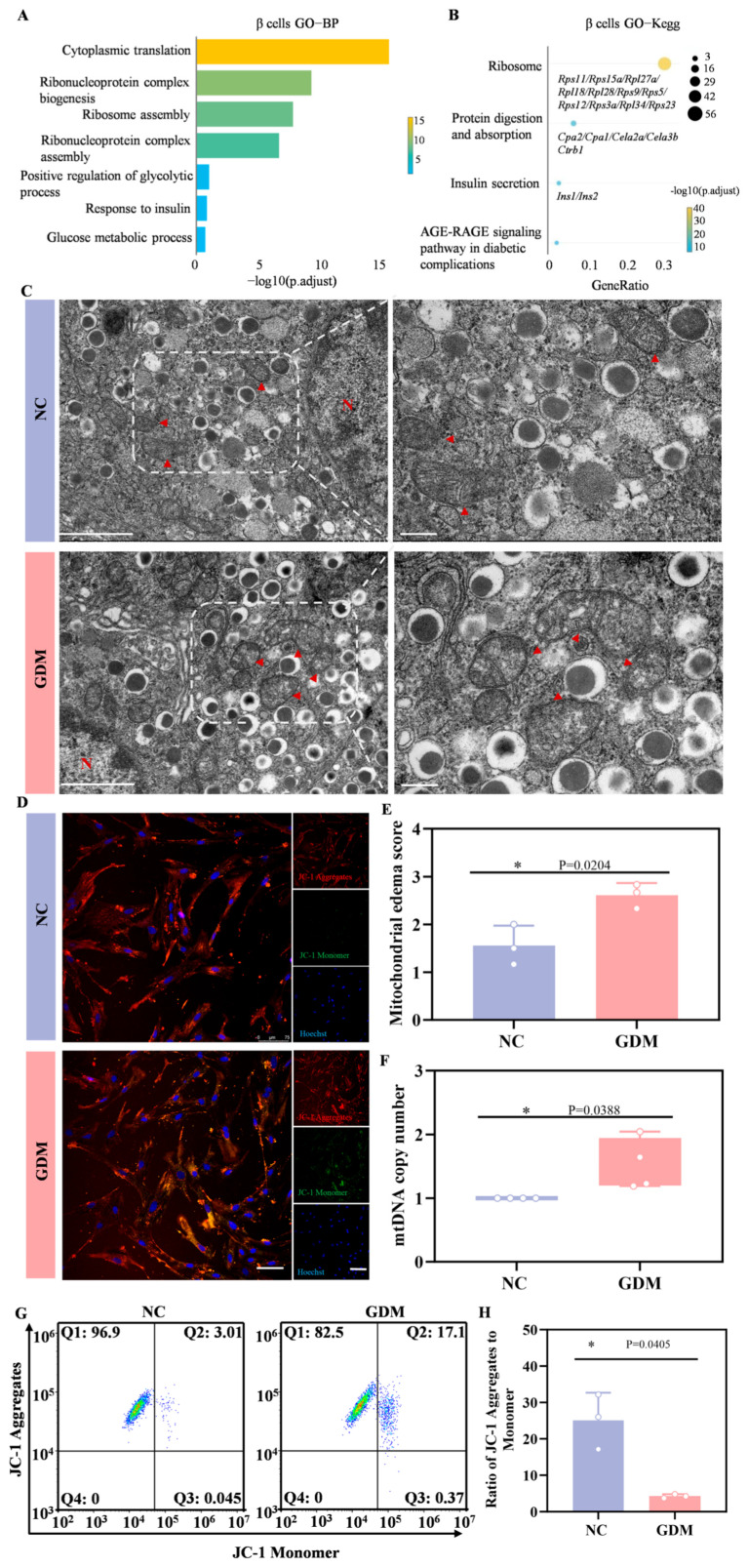
WD-induced GDM triggers a heightened stress response in the β cells of P0 offspring rats. (**A**) GO enrichment of DEGs in the biological processes of β cells. (**B**) KEGG pathways enriched by upregulated DEGs in β cells of the GDM group compared with the control group. (**C**) TEM of β cells in offspring rats (left, Scale bar, 5 μm), and locally enlarged image (right, Scale bar, 1 μm). The red arrow points to the mitochondria (n = 3 L). (**D**) Representative confocal images of JC-1 staining in primary islet cells from offspring rats (red indicates aggregates, green indicates monomers). (**E**) Mitochondrial edema score. (**F**) The copy number of mtDNA in islets of offspring rats (n = 3 L). (**G**) Representative dot plots of JC-1 staining in primary islet cells from offspring rats (n = 3 L). (**H**) Quantitative analysis of the ratio of JC-1 aggregates to monomers. Data were analyzed using unpaired two-tailed Student’s *t* tests. Results are expressed as mean ± SEM, with statistical significance indicated as follows: * *p* < 0.05.

Subsequently, we performed Gene Ontology (GO) enrichment analysis and Kyoto Encyclopedia of Genes and Genomes (KEGG) pathway analysis on the differentially expressed genes (DEGs) in β cells. Figure 6A showed that gene sets related to cytoplasmic translation, ribonucleoprotein complex biogenesis, ribosome assembly, glycolytic process, and glucose metabolic process were significantly enriched. KEGG pathway analysis further revealed that in the β cells of WD-induced GDM-offspring rats, the expression of genes involved in ribosome function, protein digestion and absorption, and insulin secretion-related signaling pathways was significantly upregulated (Figure 6B). These results suggested that the aforementioned signaling pathways were significantly activated in early β cells of WD-induced GDM-offspring rats.

In ultrastructural analyses, TEM revealed abnormal mitochondrial morphologies, including matrix edema and reduced cristae in the β cells from WD-induced GDM-offspring islets, relative to the NC group (Figure 6C,E). Quantitative analyses further showed a significant increase in mtDNA copy number in β cells from the offspring of WD-induced GDM rats (Figure 6F). As depicted in Figure 6D, most mitochondria in islet cells from WD-induced GDM offspring exhibited reduced membrane potential (indicated by green fluorescence), whereas those in the NC group maintained a normal potential (indicated by red fluorescence). Flow cytometry analysis of JC-1 staining also yielded consistent results (Figure 6G,H). Notably, our previous work confirmed prominent endoplasmic reticulum stress in islet β cells of P0 GDM offspring. These indicate that significant metabolic stress and organelle dysfunction risks underlie the compensatory activation.

## 4. Discussion

Epidemiological studies reveal that approximately 43% of pregnant women experience excessive gestational weight gain [28], and among women with GDM, the proportion of pre-pregnancy overweight or obesity is as high as 67% [29]. HFD, especially the WD rich in saturated fats and cholesterol, is one of the key environmental factors inducing maternal obesity. Long-term HFD intake leads to energy surplus, which in turn causes adipose tissue expansion, increased circulating free fatty acid levels, and elicits systemic low-grade inflammation. These metabolic abnormalities further interfere with insulin signaling, promoting the development of insulin resistance and compensatory hyperinsulinemia. During pregnancy, this metabolic imbalance can be directly transmitted to the developing fetus through the placental interface, thereby forming a GDM intrauterine microenvironment characterized by hyperglycemia, hyperlipidemia, and elevated inflammatory factor levels.

The islets of Langerhans are a crucial tissue for maintaining systemic glucose homeostasis. Among them, α cells secrete glucagon, while β cells produce insulin—the only hormone in the body that lowers blood glucose. Notably, these cells are extremely sensitive to changes in energy status, and abnormalities in the intrauterine metabolic environment may interfere with the process of their normal differentiation and functional establishment. Animal studies have demonstrated that fetal mice exposed to hyperglycemia during late gestation [30] or throughout pregnancy [31] exhibit significantly reduced insulin sensitivity, coupled with suppressed expression of key β-cell transcription factors PDX1 and MafA. More critically, such dysregulation may trigger intergenerational metabolic programming via nutrient-mediated and epigenetic mechanisms. On one hand, multiple studies indicate that metabolic disturbances in GDM mothers alter fetal metabolism of nutrients such as amino acids and fatty acids, subsequently affecting energy metabolism and insulin sensitivity in offspring [32,33]. On the other hand, maternal hyperglycemia alters methylation-associated molecules in oocytes (e.g., Tet3, EZH2, DNMT1), resulting in aberrant DNA methylation patterns in offspring [34,35,36]. This epigenetic dysregulation subsequently triggers insufficient insulin secretion, causes glucose intolerance, and potentially drives intergenerational transmission of metabolic disorders.

As our previous study indicated, the differences in islet phenotypes between the control and GDM offspring were most pronounced at P0 [16,17]. Therefore, in the current study, we focus on the impact of WD-induced GDM on the function of islets in their offspring, comprehensively characterize the developmental abnormalities emerging at P0, and elucidate their underlying molecular mechanisms. Given that a WD more closely recapitulates modern human dietary patterns than a 60% HFD, we employed this diet to successfully establish a GDM model with concomitant insulin resistance in pregnant rats. Maternal WD exposure resulted in disrupted glucose homeostasis in offspring as early as P0, characterized by concomitant hypoglycemia and hyperinsulinemia. This phenomenon may result from the intrauterine hypermetabolic microenvironment, which stimulates compensatory oversecretion of insulin by offspring β cells. After birth, upon exposure to a normoglycemic environment, this persistent hypersecretion of insulin manifests as fasting hypoglycemia. This “hyperinsulinemia-hypoglycemia” state directly reflects the excessive development and hypercompensation of offspring islets under intrauterine metabolic stress. Immunofluorescence staining revealed compensatory hyperplasia in the islets of offspring exposed to the WD. Furthermore, in vitro insulin secretion assays demonstrated that islets from this group of offspring not only responded to amino acid stimulation by secreting insulin but were also capable of sensing high-glucose signals and secreting insulin, whereas normal neonatal islets typically exhibit insulin secretion only in response to amino acids [20]. These results indicate that under the metabolic stress induced by maternal WD exposure, the offspring islets have mounted a robust adaptive response. To decipher the molecular basis of these developmental anomalies, we performed scRNA-seq on P0 offspring islets. The results further demonstrated that in WD-induced GDM offspring, there was a significant increase in endocrine cell proportions and pronounced dysregulation of α/β-cell subsets, specifically characterized by a marked reduction in immature α1 and β1 subsets versus a substantial elevation in mature α2, β2, β3, and β4 subsets. Collectively, these findings indicated that the offspring developed prenatally established islet impairments under maternal WD exposure.

Gene Set Enrichment Analysis (GSEA) showed that significant upregulation of PI3K-Akt and AMPK signaling pathways in WD-induced GDM offspring islets, consistent with our prior discovery that “GDM activates the PI3K-Akt-FoxO1/mTORC1 axis via exosomal miR-7-19488 from umbilical vein blood” [17]. This coherence strongly suggested that aberrant PI3K-Akt activation might constitute a central pathological event.

Furthermore, sequencing and immunofluorescence results demonstrated that in the α cells of WD-induced GDM offspring, GLP-1 biosynthesis was enhanced. This finding suggests that under maternal metabolic stress, offspring islets may activate an endogenous GLP-1 synthesis pathway. Although circulating active GLP-1 primarily originates from intestinal L cells, it is rapidly degraded by dipeptidyl peptidase-4 (DPP-4) after secretion, leaving only a small residual amount of active GLP-1 to reach and regulate islets via the bloodstream. During embryonic development, due to the absence of oral intake, stimulation of intestinal L cells is minimal, and circulating GLP-1 levels are likely limited. Recent studies have suggested that a more significant local GLP-1 signaling axis may exist within the islets, especially during development. For instance, transient expression of PCSK1 in α cells and generation of active GLP-1 have been observed in embryonic pig islets, while developmental stage-specific changes in GLP1R and DPP4 expression in β cells collectively support the existence of an intra-islet autocrine/paracrine GLP-1 signaling system [24]. Given our earlier proposition that “local GLP-1/GLP-1R axis activation further stimulates PI3K-Akt signaling” [17], we propose that under metabolic stress, offspring islets may engage in adaptive regulation by activating endogenous GLP-1 synthesis. This could represent a compensatory response of embryonic islets to high-energy load, though its long-term effects and functional sustainability require further investigation.

What precisely is the functional role of the AMPK pathway in this process? KEGG pathway analysis revealed upregulation of genes associated with ribosomal function and protein digestion/absorption in WD-induced GDM-offspring β cells. As a central energy-sensing hub, AMPK signaling likely contributes to the metabolic adaptation of islet cells by regulating these processes. Specifically, under intrauterine hyperglycemia, AMPK activation enhances cellular energy-status perception and cooperates with the PI3K-Akt pathway to initiate molecular mechanisms governing insulin synthesis and secretion—thereby driving islets into a premature hypersecretory “quasi-mature state”.

Is this “quasi-mature state” truly beneficial to islets? Our results demonstrated that the hypersecretory phenotype in WD-induced GDM-offspring β cells comes at the cost of organelle dysfunction, manifested as mitochondrial matrix edema, cristae reduction, and heightened endoplasmic reticulum stress (ER). Delving into the mechanistic level, this phenotype may involve a vicious cycle of mitochondrial dysfunction and oxidative stress. In a hyperglycemic environment, excessive reactive oxygen species (ROS) can be generated in the mitochondria of β cells [37]. This oxidative stress directly damages mitochondrial DNA and protein complexes, impairs electron transport chain efficiency, and reduces membrane potential—consistent with the observations in this study [38]. More critically, dysfunctional mitochondria further generate increased ROS, and the accumulated ROS can disrupt protein folding in the ER, exacerbating ER stress. Conversely, ER stress can also impair mitochondrial function through mechanisms such as calcium dysregulation, thereby establishing a vicious cycle along the “mitochondria–ER stress axis [39].” This inter-organelle cross-damage mechanism may create a form of “metabolic memory” in β cells, rendering them more susceptible to accelerated functional decline when facing future metabolic challenges. Concurrently, this study revealed significant enrichment of ribosomal-related pathways in β cells, suggesting that cells attempt to cope with metabolic stress by enhancing protein synthesis. Given that protein synthesis is highly dependent on mitochondrial ATP supply, abnormally elevated ribosomal activity inevitably exacerbates mitochondrial energy burden. The TEM results provided direct morphological evidence of mitochondrial swelling and ER dilation. Essentially, this state represents an adaptive compensation by β cells to intrauterine hypermetabolic stress. Short-term homeostasis is maintained through hyperfunctional overload, yet persistent organelle damage—particularly in mitochondria and ER—plants the seeds for future functional decompensation.

This study also has several limitations. First, our structural conclusions from TEM are based on a limited number of samples (n = 3 L) and should be interpreted with caution. Second, we focused solely on the time point of the day of birth (P0). While this allows us to reveal significant phenotypes at a critical window, it does not provide a complete picture of the dynamic trajectory of islet development. Third, as the external genitalia of newborn rats are not yet distinctly differentiated, we did not perform sex-distinguished analyses in the experiments. This may obscure potential sex-specific responses, and future studies could incorporate earlier sex determination methods for refinement. Finally, the tissue dissociation process required for ScRNA-seq may introduce non-specific effects on cellular states and gene expression. Although we implemented standardized protocols and strict quality controls and focused on consistent changes at the pathway level in our analyses, this technical limitation must still be considered when interpreting the results.

“Ten months of pregnancy culminate in childbirth,” a period of intricate biological cross-talk between mother and fetus that orchestrates fetal development. This gestational window represents a critical phase of developmental programming, wherein maternal nutrition, especially in the context of overnutrition, directly dictates long-term metabolic trajectories. Due to the limitations regarding the safety of medication during pregnancy, a healthy lifestyle is particularly important throughout the entire pregnancy. A high-fat and high-sugar diet should be avoided, especially for pregnant women who are of advanced age, obese, or have a family history of diabetes. Moving forward, we will continue to investigate the key molecular mediators within the GDM microenvironment that link maternal overnutrition to aberrant islet development, with the ultimate goal of developing nutrient-based early interventions to mitigate the intergenerational transmission of metabolic disease.

## 5. Conclusions

This study demonstrates that maternal Western diet-induced GDM reprograms offspring islet development, leading to accelerated α/β-cell maturation and hyperinsulinemia at birth. However, this adaptive response causes significant β-cell organelle damage, including mitochondrial dysfunction and endoplasmic reticulum stress. These findings highlight how maternal metabolic disturbances during pregnancy can disrupt offspring pancreatic health, supporting the need for early nutritional interventions to prevent intergenerational transmission of diabetes.

## Data Availability

The materials used in this study are described in Section 2, Materials and Methods. The results of single-cell RNA sequencing have been deposited in the GEO database.

## Abbreviations

The following abbreviations are used in this manuscript:

GDM	Gestational diabetes mellitus
ScRNA-seq	Single-cell RNA sequencing
OGTT	Oral glucose tolerance test
Sst	Somatostatin
DOHaD	Developmental Origins of Health and Disease
T2D	Type 2 diabetes
PGDM	Pregestational diabetes mellitus
NC	Normal control
E0.5	Embryonic day 0.5
P0	Postnatal day 0
IF	Immunofluorescence
TEM	Transmission electron microscope
FBS	Fetal bovine serum
KRB	Krebs Ringer Bicarbonate
AA	Amino acid
MtDNA	Mitochondrial DNA
ΔΨm	Mitochondrial membrane potential
AUC	Area under the blood glucose curve
HOMA-IR	Homeostatic Model Assessment of Insulin Resistance
Gcg	Glucagon
Ins	Insulin
GLP-1	Glucagon-like peptide-1
